# Maternal bacteria to correct abnormal gut microbiota in babies born by C-section

**DOI:** 10.1097/MD.0000000000021315

**Published:** 2020-07-24

**Authors:** Éadaoin M. Butler, Valentina Chiavaroli, José G.B. Derraik, Celia P. Grigg, Brooke C. Wilson, Nicholas Walker, Justin M. O'Sullivan, Wayne S. Cutfield

**Affiliations:** a A Better Start – National Science Challenge; b Liggins Institute, University of Auckland, Auckland, New Zealand; c Neonatal Intensive Care Unit, Pescara Public Hospital, Pescara, Italy; d Department of Women's and Children's Health, Uppsala University, Uppsala, Sweden; e Endocrinology Department, Children's Hospital of Zhejiang University School of Medicine, Hangzhou, China; f Department of Obstetrics and Gynaecology, Auckland City Hospital, Auckland District Health Board, New Zealand.

**Keywords:** caesarean section, microbiome, obesity, vaginal bacteria

## Abstract

**Introduction:**

There is evidence that caesarean section (CS) is associated with increased risk of childhood obesity, asthma, and coeliac disease. The gut microbiota of CS-born babies differs to those born vaginally, possibly due to reduced exposure to maternal vaginal bacteria during birth. Vaginal seeding is a currently unproven practice intended to reduce such differences, so that the gut microbiota of CS-born babies is similar to that of babies born vaginally. Our pilot study, which uses oral administration as a novel form of vaginal seeding, will assess the degree of maternal strain transfer and overall efficacy of the procedure for establishing normal gut microbiota development.

**Methods and analysis:**

Protocol for a single-blinded, randomized, placebo-controlled pilot study of a previously untested method of vaginal seeding (oral administration) in 30 CS-born babies. A sample of maternal vaginal bacteria is obtained prior to CS, and mixed with 5 ml sterile water to obtain a supernatant. Healthy babies are randomized at 1:1 to receive active treatment (3 ml supernatant) or placebo (3 ml sterile water). A reference group of 15 non-randomized vaginal-born babies are also being recruited. Babies’ stool samples will undergo whole metagenomic shotgun sequencing to identify potential differences in community structure between CS babies receiving active treatment compared to those receiving placebo at age 1 month (primary outcome). Secondary outcomes include differences in overall gut community between CS groups (24 hours, 3 months); similarity of CS-seeded and placebo gut profiles to vaginally-born babies (24 hours, 1 and 3 months); degree of maternal vaginal strain transfer in CS-born babies (24 hours, 1 and 3 months); anthropometry (1 and 3 months) and body composition (3 months).

**Ethics and dissemination:**

Ethics approval by the Northern A Health and Disability Ethics Committee (18/NTA/49). Results will be published in peer-reviewed journals and presented at international conferences.

**Registration:**

Australian New Zealand Clinical Trials Registry (ACTRN12618000339257).

## 1 Introduction

### 1.1 Background and rationale

Caesarean section (CS) is a surgical intervention intended to reduce risks to the health of women and babies in cases where vaginal birth is considered unsafe. There has been a dramatic worldwide increase in rates of CS; in 1990 fewer than 1 in 10 women gave birth in this manner, whereas by 2014 this figure was closer to 1 in 5.^[1]^ In New Zealand, about 25% of babies are born by CS.^[2]^

A recent meta-analysis of long-term benefits and risks associated with CS reported reduced rates of urinary incontinence and pelvic organ prolapse in women giving birth at term.^[3]^ However, it also highlighted a range of other increased health risks to both mother (e.g., subsequent stillbirth, placenta previa, and placenta accreta) and child (e.g., childhood obesity).^[3]^ Indeed, a 2015 meta-analysis reported a risk ratio of 1.29 for obesity in offspring born by CS, compared to those born vaginally.^[4]^ Since then, a number of other studies have also reported increased risk of obesity in children born by CS,^[5–8]^ although not consistently.^[9]^ Of note, a US study of over 22,000 participants found a 64% increased risk of obesity for those born by CS compared to their vaginal-born siblings.^[10]^ This increased risk may be established early. One study detected accelerated weight gain among infants born by CS as soon as 3 months,^[11]^ while another reported higher mean body mass index (BMI) at 6 months, although this difference was not observed in later childhood.^[12]^ Aside from obesity, there is also inconsistent evidence for an association between birth by CS and increased risk of asthma,^[13–18]^ eczema/atopic dermatitis,^[16,18]^ coeliac disease,^[19–22]^ and type 1 diabetes.^[23–26]^

Altered colonization of the gut microbiota in babies born by CS may partially account for the increased risk of obesity and other health conditions. For many years, the sterility of the womb was accepted as fact.^[27]^ In recent years, evidence has emerged of a placental microbiota,^[28–30]^ although this remains controversial, and even if in existence, its biological significance remains speculative.^[31]^ Birth is an important process for the initial colonization of the gut microbiota. Babies born vaginally are exposed to their mother's vaginal microbes during birth. These microbes are likely to be dominated by species of *Lactobacillus,* key vaginal colonizers,^[32]^ particularly during pregnancy.^[33,34]^ Previous research of 178 mother-baby dyads showed that maternal microbial strains were much more frequently transmitted to the gut microbiota of babies born vaginally during the neonatal period than CS-born babies.^[35]^ Neonates’ stomachs are pH neutral for several hours post-birth due to the amniotic fluid they swallow in utero,^[36]^ thereby enabling survival of bacteria ingested during birth. The early gut microbiota of vaginal-born babies is dominated by species of commensal bacteria such as *Bifidobacterium* and *Bacteroides*,^[35]^ which are associated with improved immune function and reduced inflammation.^[37]^

Babies born by elective CS do not encounter maternal vaginal microbes, while there is conflicting evidence as to whether this is the case for babies born by emergency CS.^[35,38]^ Overall, the early gut microbiota of babies born by CS is dominated by typical hospital and skin colonisers, for example, *Staphylococcus*^[35]^ and *Streptococcus*.^[39]^ In fact, gut colonization by *Bacteroides* has been shown to be delayed up to one year for CS-born babies.^[40,41]^ Interestingly, the gut microbiota of 1-week old CS-born babies has been found to have increased abundance of Firmicutes and decreased abundance of Bacteroidetes compared to vaginal-born babies, although no such differences were seen by 8 or 24 weeks of age.^[42]^*Bifidobacterium* numbers are reduced in the early gut microbiota of children above a healthy weight by 7-years of age,^[43]^ as is also the case in the early gut microbiota of CS-born babies.^[35]^ Therefore, given that dysbiosis (imbalance) of the gut microbiota has been linked to obesity,^[44]^ it is interesting to speculate that impairment in normal microbiota development in CS-born babies is linked to the observed phenotypic changes later in life.

Vaginal seeding (exposure of CS-born babies to maternal vaginal fluids after birth) is a potential mechanism for reducing differences in the gut microbiota of CS-born and vaginal-born babies. In the US, a small pilot study orally and topically swabbed CS-born babies (n = 4) with their mothers’ vaginal microbes shortly after birth.^[45]^ At 1 month of age, these babies had skin, oral, and anal microbiota more similar to those of vaginal-born babies than CS-born babies who were not swabbed.^[45]^ While these findings are encouraging, no baby stool samples were collected so it remains unclear what effect (if any) the seeding had on the babies’ gut microbiota. It is also possible that such swabbing may not be the most optimal method of seeding a baby's gut microbiota; oral administration for example, while previously untested, may be more effective. Further, concerns regarding potential transmission of infectious diseases to neonates has prompted the American College of Obstetricians and Gynecologists to recommend that vaginal seeding only be performed within the context of an approved research protocol until more data regarding safety and benefit are available.^[46]^ Considering the above, it is clear that further research is warranted regarding this promising, but unproven practice.

We describe here the protocol for the ECOBABe (Early COlonization with Bacteria After Birth) pilot study, a prospective, single-blinded, placebo-controlled trial, which will assess the effectiveness of a novel method of vaginal seeding (oral administration) for altering the gut community of CS-born babies. Thirty CS-born babies are randomized to be orally administered active treatment (3 ml vaginal bacteria supernatant) or placebo (3 ml sterile water). The study also includes a reference group of non-randomized vaginal-born babies to enable comparison of microbiota development observed in CS-born babies to that of vaginal-born babies. All eligible babies (randomized CS-born and non-randomized vaginal-born) will be followed up to three months after birth. We have followed the SPIRIT guidelines in the reporting of this protocol.^[47]^

### 1.2 Objectives

The objectives of this pilot RCT will be to explore if a previously untested method of vaginal seeding (oral administration) will: i) alter the overall gut community in seeded CS-born babies; and ii) alter the gut profiles of these babies to more closely resemble the gut microbiota of babies born vaginally. If oral administration proves successful, a larger study with longer follow-up will be warranted.

## 2 Methods

### 2.1 Study setting

The study is assessing the eligibility of pregnant women planning an elective CS or vaginal birth across the entire Auckland region of Aotearoa/New Zealand, where over 20,000 women gave birth in 2017.^[2]^ Maternal vaginal bacteria or placebo is administered to CS-born neonates in hospital theatres shortly after birth. Follow-up of most babies occurs at the Maurice and Agnes Paykel Clinical Research Unit, Liggins Institute, University of Auckland. Some one-month follow-ups occur at a facility closer to, or at, the participant's home (where onsite visit is not practicable).

### 2.2 Eligibility criteria

Babies are stratified into one of 2 groups: randomized CS-born or non-randomized vaginal-born. Eligibility assessments occur as a two-step process for each group; the first assessments are antenatal and the second occur at birth. Table 1 outlines exclusion criteria at each step for mothers and babies in the CS group. All pregnant women planning to have an elective CS undergo study-specific antenatal screening no earlier than 10 days before their scheduled CS. This is performed by a member of the research team at our clinical research unit or at a location more convenient for the participant (e.g., local maternity unit or home). All CS group antenatal screening samples are analyzed at Middlemore Hospital Laboratory, Auckland. Table 2 outlines exclusion criteria at each step (antenatal and birth) for those in the vaginal birth reference group.

**Table 1:**
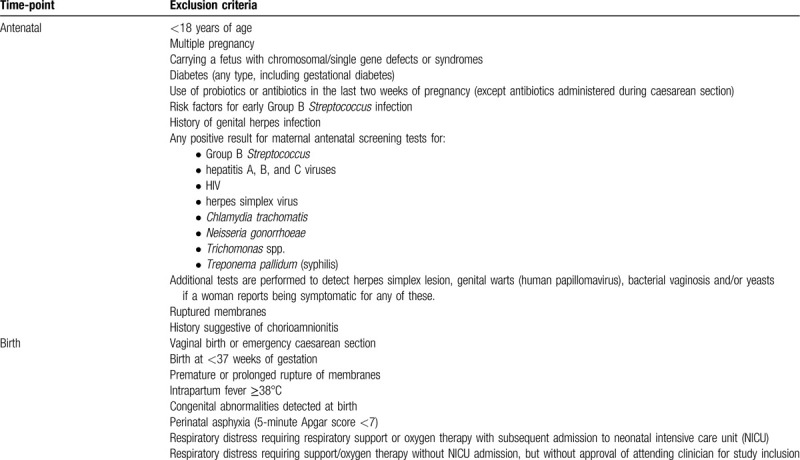
Exclusion criteria for mothers and babies in the caesarean section group.

**Table 2:**
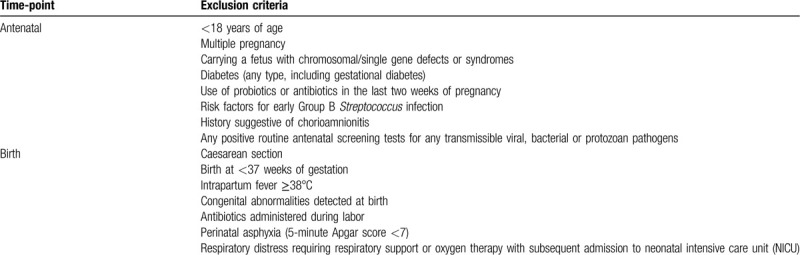
Exclusion criteria for mothers and babies in the vaginal birth group.

### 2.3 Interventions

Eligible babies in the elective CS group are randomized in a 1:1 ratio to receive either active treatment (3 ml vaginal bacteria supernatant) or placebo (3 ml sterile water). A folded 13 × 300 mm sterile gauze with an x-ray strip (Propax, New Zealand) is inserted into the lower vagina of women in the CS group and removed after approximately 30 minutes, as close as possible to their surgery. Women can choose to insert and/or remove the gauze themselves, or have these tasks performed by a clinical member of the research team. Sterile gloves are worn during insertion and removal of the gauze. A counter-signed study swab-count sticker is used to verify the removal of the swab prior to surgery, and added to the woman's clinical notes.

After removal, the gauze is vertically cut into two equal sized pieces using sterile scissors. One piece is kept on ice in a sterile container for transportation back to the Liggins Institute laboratory. The other piece is placed in a sterile syringe with 5 ml of sterile water and mixed to obtain a vaginal bacteria supernatant. Next, 3 ml of this supernatant is transferred to a 3 ml sterile syringe in preparation for oral administration to babies randomized to receive active treatment. The remaining 2 ml of supernatant are transferred to a microcentrifuge tube and stored on ice for transportation back to the laboratory, for later microbiota assessment. If a baby is randomized to receive placebo, only 3 ml of sterile water is placed in the syringe, while the prepared vaginal supernatant is reserved solely for microbiota assessment.

If the attending clinician assesses the newborn as healthy, a member of the research team uses the pre-filled syringe to orally administer either active treatment (3 ml vaginal bacteria supernatant) or placebo (3 ml sterile water). Administration occurs as soon as possible after birth (no later than one hour). Babies in the vaginal-born reference group are not randomized, as no administration of active treatment or placebo occurs in this group.

### 2.4 Outcomes

Primary outcome:

- Difference in the community structure of the gut microbiota between CS groups at 1 month of age.

Secondary outcomes:

- Difference in the overall gut microbiota community between CS groups at 24 hours and 3 months of age.
- Similarity of CS seeded and placebo gut microbiota profiles to those born vaginally at 24 hours, 1 and 3 months of age.
- Degree of maternal vaginal bacterial strain transfer in CS-born babies at 24 hours, 1 and 3 months of age.
- BMI *z*-score at 1 and 3 months of age.
- Total body fat percentage from whole-body dual-energy absorptiometry (DXA) at 3 months of age.

### 2.5 Participant timeline

**Figure 1:**
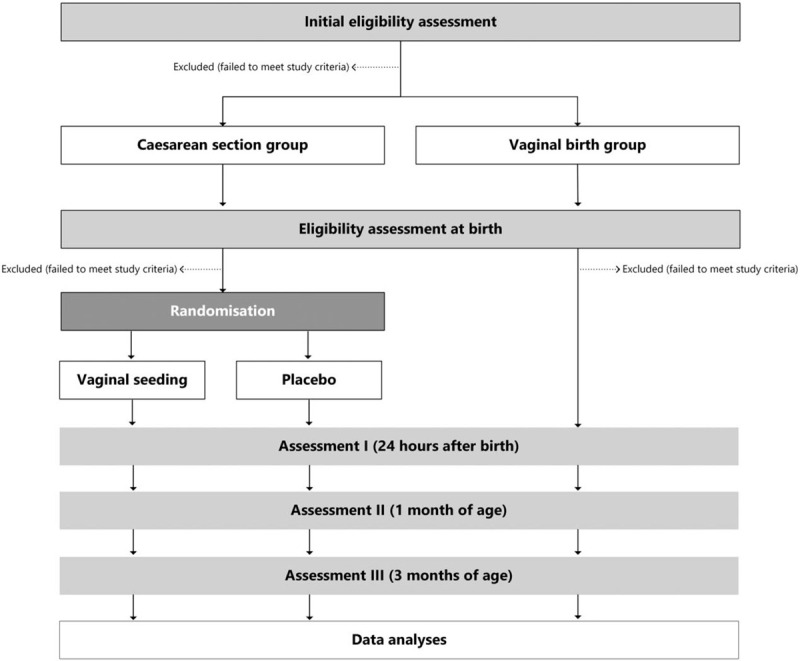
Flow of participants through the ECOBABe study.

Pregnant women in the CS group are screened no earlier than 10 days before their scheduled CS. Participants in both groups complete 3 online or paper antenatal questionnaires approximately 2 weeks prior to their estimated due date. The mothers and their babies are followed for three months post birth. Figure 1 shows the flow of participants in both the vaginal and CS birth groups.

### 2.6 Sample size

We intend to randomize 30 healthy babies in the CS group on a 1:1 basis to intervention or placebo (15 per group). A power calculation indicated that 15 babies per CS group would be sufficient to detect a moderate effect size of 0.25 standard deviations for our primary outcome (power calculation for balanced one-way ANOVA test, 85% power, α = 0.05). In addition, we intend to recruit 15 babies born vaginally as a reference group.

### 2.7 Recruitment

Recruitment occurs in conjunction with midwives and obstetricians working throughout the Auckland region. In addition, targeted recruitment posts are placed on social media. Posters and study leaflets are also distributed across Auckland to places where pregnant women are likely to see them, for example, public and private obstetric clinics, midwifery clinics, and antenatal education classes. In addition, the research team attended a popular local pregnancy and early childhood fair, and spoke with interested pregnant women present.

Woman exposed to recruitment material may self-refer to the study by emailing, texting, or calling the research team, or through completion of a brief online form on the study webpage. With the permission of interested women, health professionals can also pass on their contact details to the study team. The research team is responsible for obtaining informed consent from all potential participants.

### 2.8 Randomization, allocation, and blinding

Healthy CS-born babies are randomized in a 1:1 ratio to either treatment or placebo group, using computer-generated randomization. Mothers of randomized babies were blinded to group allocation; the researchers analyzing the primary outcome data, are blinded to baby group (active treatment, placebo, or vaginal-born reference group). For pragmatic reasons, it is not possible for the researcher administering the intervention to be blinded, as they are the only member of the research team present at a woman's CS. Participant un-blinding during the study is permissible in the case of any serious adverse events and upon recommendation by the study's independent data monitoring committee (DMC).

### 2.9 Data collection and follow-up

#### 2.9.1 Timing of assessments

Babies are assessed at three time-points over the course of the study: birth (24 hours), 1 month, and 3 months (Table 3). All mothers are assessed for eligibility in the antenatal period, with just postnatal maternal health issues recorded thereafter (Table 3). Mothers are reminded of their study visits via email or text message. Fuel vouchers and free parking are provided for all study visits, and taxis are booked for mothers who are unable to drive to appointments. Study visits are rescheduled if a mother indicates that she will be unable to attend at the booked time.

**Table 3:**
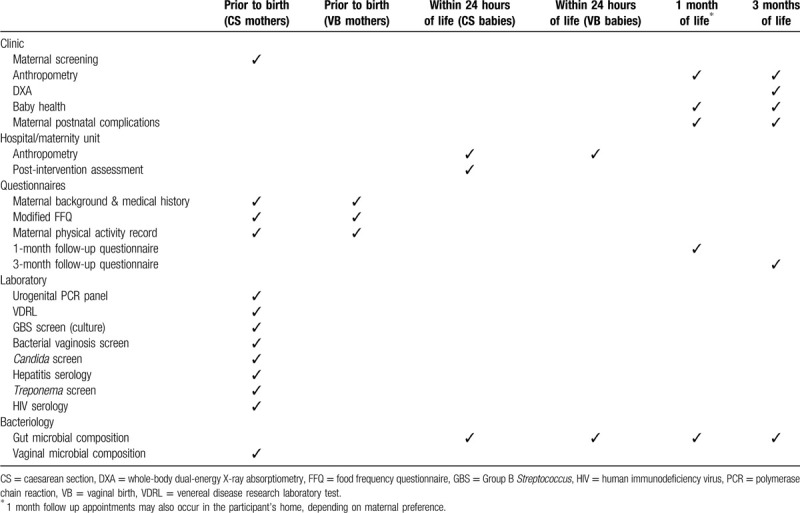
Timing of assessments for mothers and babies in the ECOBABe study.

#### 2.9.2 Maternal dietary intake

All women report their antenatal dietary intake using a modified version of The New Zealand Adolescent Food Frequency Questionnaire (NZAFFQ).^[48]^ The questionnaire asks respondents to rate the frequency with which they consume specific types of food and fluids. No food frequency questionnaire (FFQ) has yet been validated for pregnant women in New Zealand. While FFQs have been validated for New Zealand adults,^[49,50]^ they include questions regarding alcohol (which pregnant women are advised against consuming). Thus, we are using a modified version of the NZAFFQ, which does not include such questions. Nevertheless, potential maternal alcohol intake during pregnancy is recorded on a maternal background and medical history questionnaire (below).

#### 2.9.3 Maternal physical activity

The women report their antenatal physical activity using the short form of the International Physical Activity Questionnaire (IPAQ).^[51]^ All women are asked to recall their physical activity levels for the previous seven days.

#### 2.9.4 Maternal background, medical history, and anthropometry

The women complete a brief questionnaire regarding their:

- demographic information: date of birth, ethnicity, place of birth, education
- medical history: serious health problems, medical treatments, family history of medical problems, previous pregnancies
- probiotic supplement intake
- current pregnancy information: conception type, illness during pregnancy, smoking and alcohol consumption during pregnancy
- current participation in other clinical trials
- pre-pregnancy weight and height.

#### 2.9.5 Neonatal assessments

Neonatal assessments differ according to participant study group (CS vs vaginal birth). Post-treatment neonatal clinical assessments are performed only on babies born by CS. A member of the study team records birth weight, length, head circumference, abdominal circumference, and chest circumference measurements for all CS-born babies. These measurements are taken using the weighing scales and tape measures in the hospital where participants give birth. Other neonatal clinical information is also recorded by a research clinician for CS-born babies (e.g., Apgar scores, breastfeeding initiation, and skin-to-skin start time).

Neonatal clinical information for vaginal-born babies are obtained from medical records. Note that abdominal and chest circumference measurements at birth are not recorded for vaginal-born babies as these are not standard measurements, and it is not feasible to have a member of the study team present at these births.

#### 2.9.6 Baby anthropometry and body composition

At 1 and 3 months of age, all babies are weighed and measured while naked. They are weighed using an infant weighing scale. Crown-heel length is recorded three times using a neonatometer (Holtain Ltd., Crymych, UK) with gentle pressure applied to the legs to ensure the body is flat as possible. The median value of the three measurements will be used for analysis. Both the weighing scale and neonatometer are regularly calibrated. Head, abdominal, and chest circumferences are measured using disposable paper measuring tapes. At 3 months, baby body composition is assessed using DXA while wrapped in a blanket (Lunar Prodigy and Lunar iDXA, GE Medical Systems, Chicago, IL).

#### 2.9.7 Sample collection and processing

Baby stool samples are collected by the parents from fresh nappies within the first 24 hours of life, and again at 1 and 3 months of age. Approximately one gram of stool is collected using the scoop attached to the lid of a sterile specimen tube (Sarstedt, Nümbrecht, Germany, Catalogue #SARS80.623). The tubes are prefilled with 5 ml of DNA/RNA Shield^TM^ solution (Zymo Research, Irvine, California, US, Catalogue #R1100) enabling them to be kept stable at room temperature until transfer to the laboratory. Parents are asked to shake the tightly-closed tube to mix the stool with the solution immediately after collection. Parents record the date and time of sample collection on an accompanying collection card. Upon arrival at the laboratory, stool samples are split into 1 ml aliquots and stored at −80°C.

Maternal vaginal microbiota samples (2 ml of vaginal bacteria supernatant and the remaining half of the gauze swab) are collected from all mothers in the CS group just prior to their scheduled CS, and are kept on ice until transfer to −80°C.

Microbial DNA will be extracted using the ZymoBIOMICS^TM^ 96 MagBead DNA kit (Catalogue #D4308) according to the manufacturer's instructions. A blank DNA extraction control consisting of 1 ml of sterile water will be run in parallel for contamination testing. In addition, the ZymoBIOMICS^TM^ microbial community standard (Catalogue #D6300) will serve as an extraction bias control. DNA extracts will be quantified using the Qubit^TM^ dsDNA High Sensitivity Assay kit (Catalogue #Q32851) with a Qubit^TM^ 3.0 fluorometer. Whole metagenome shotgun sequencing will be performed by an independent accredited commercial provider.

#### 2.9.8 Microbial and statistical analyses

Metagenomic sequencing data will be processed with bioBakery workflows using docker images available at http://huttenhower.sph.harvard.edu/biobakery_workflows. Briefly, sequence data quality control, including removal of any human reads, will be conducted using KneadData. Taxonomic and functional profiles of the microbiota will be generated using MetaPhlAn v2.6^[52]^ and HUMAnN2^[53]^ respectively. Strain-level taxonomic profiling will be achieved by SNP haplotype based profiling using StrainPhlAn software.^[54]^ The resulting SNP haplotypes will be used to identify the proportion of shared and unrelated strains between mother-baby pairs, comparing these proportions across treatment groups.

Permutational multivariate analysis of variance (PERMANOVA) will be used to identify any significant differences in the overall microbial community structure between treatment groups. Additionally, Multivariate Association with Linear Models (MaAsLin2) will be used to identify any significant associations between specific microbial taxa and treatment groups. Both taxonomic and functional profiles will be compared. Microbiota stability across time points will be assessed using Bray Curtis dissimilarity. Microbial alpha diversity will be assessed using Shannon's diversity index and gene counts.

Treatment evaluation on clinical outcomes will be performed on the basis of intention to treat, using data collected from all randomized participants. Generalized linear regression models will be used to assess treatment effects, adjusting for sex. Model-adjusted estimates and the difference among the three groups will be calculated and tested. Planned subgroup analysis by sex may be conducted on secondary outcomes to evaluate the consistency of possible treatment effects in males and females. Analyses of non-microbiome data will be performed in SAS v9.4, SPSS v26, and/or Minitab v16. Statistical tests will be two-tailed and significance maintained at 5% level.

#### 2.9.9 Patient and public involvement

Public input into the study design was mainly provided in open meetings by members of the Northern A Health and Disability Ethics Committee, including both clinical and lay persons, as well as Māori representatives (indigenous people of New Zealand). In addition, we have consulted with a number of healthcare practitioners in the early stages, so that feedback from obstetricians, midwives, and lactation consultants were incorporated whenever possible. However, participants have not been involved in the development or conduct of the trial.

### 2.10 Data management

Data are entered into Research Electronic Data Capture (REDCap)^[55,56]^ hosted at the University of Auckland. REDCap is a secure password-protected web-based research platform. Human error is minimized through use of validation rules, designed to reduce the potential of incorrect data entry. Only members of the research team have access to the dataset.

### 2.11 Safety monitoring

Approximately 30 minutes post administration of either active treatment (3 ml vaginal bacteria supernatant) or placebo (3 ml sterile water), a member of the study team assesses general wellbeing, temperature, heart rate, and respiratory rate of all randomized CS-born babies. In addition, a checklist of potential adverse events has been created, and all mothers are questioned about any potential adverse events during their 1- and 3-month follow-up visits. Mothers in the CS group are advised to report any unexpected medical review/intervention for their baby to the study team as soon as possible.

An independent DMC has been established. Any potential adverse events will be recorded and reported to the DMC for their assessment. Serious adverse events (neonatal death, sepsis, intestinal obstruction, severe hyperbilirubinemia, sudden unexpected death in infancy, necrotizing enterocolitis, or maternal obstetric sepsis) will be reported to the DMC within two days of the research team being made aware of its occurrence.

If any CS-group maternal antenatal screening tests are positive, the woman will be excluded from the study and her lead maternity carer is notified so that they may take further action as appropriate. Further investigation will be immediately undertaken if any health concerns requiring referral are identified at the babies’ 1- or 3-month follow-up visits. Participants will be withdrawn from the study if this is considered to be in their best interest.

Participants are eligible to apply for compensation from the Accident Compensation Cooperation (ACC) in the event that they suffer harm due to study participation. ACC is a compulsory personal injury insurance cover for everyone in New Zealand.

## 3 Ethics and dissemination

### 3.1 Research ethics approval

Ethics approval for the study was granted by the Northern A Health and Disability Ethics Committee (reference number: 18/NTA/49). Consultation with Māori occurred via the Liggins Institute Māori Advisory Group and Ngā Māia ki Tamaki Makarau (Māori Midwives Association, Auckland Region). In addition, locality approval was granted by Auckland District Health Board Research Office (reference number: A+ 8197), Counties Manukau District Health Board Research and Evaluation Office (reference number: 976), and Waitematā District Health Board Research and Knowledge Centre (reference number: RM14063). Any subsequent amendments to the study protocol that affected study conduct were approved by the Northern A Health and Disability Ethics Committee, and notification was sent to all relevant institutions where the study is being performed.

This study is registered with the Australian New Zealand Clinical Trials Registry (ACTRN12618000339257). A Universal Trial Number (UTN), WHO, was obtained (U1111-1206-7066). The study protocol adheres to the ethical guidelines outlined in the Declaration of Helsinki. All participants provide written and oral informed consent.

### 3.2 Confidentiality

Any information collected from potential and consented participants are stored securely and can only be accessed by the research team. Hard copies of consent forms, case report forms, laboratory reports, and participant questionnaires are stored in a locked filing cabinet with access limited to research team members not involved with sample processing or analysis. Data from hard copies are subsequently entered into password-protected web-based platforms, only accessible by the research team. Participants’ study information is not released outside of the research team, with the exception of positive screening results for mothers in the CS group (as outlined above). All data and samples will be de-identified prior to analyses.

### 3.3 Dissemination policy

Findings from this study will be communicated with the scientific community and participants. Scientific communication will be through presentations at relevant international and domestic conferences and meetings, as well as publications in peer-reviewed journals. All included District Health Boards and groups with which consultation occurred will be notified of study findings through reports and/or presentations. In addition, we will communicate our findings with the general public through liaison with the Liggins Institute's communications manager.

### 3.4 Data availability

The study data cannot be made available in a public repository due to the strict conditions of the ethics approval. However, the anonymized trial data could be made available to other investigators from the corresponding author, upon bona fide request, and following all the necessary approvals (including ethics) of the detailed study proposal and statistical analyses plan.

## Acknowledgments

The authors would like to thank Dr Tommi Vatanen (Liggins Institute, University of Auckland) for his valuable input, and Janene Biggs (Liggins Institute, University of Auckland) for her ongoing support with many aspects of this study.

## Author contributions

VC, JGBD, JMOS, and WSC conceptualized and designed the study, and obtained funding; ÉMB, CPG, BCW, and NW contributed to study design. ÉMB and VC drafted the initial manuscript, which was critically revised for important intellectual content by BCW, CPG, JGBD, JMOS, NW, and WSC; all authors approved the final version of the protocol and agreed to be accountable for all aspects of the work.
